# From basics to clinic: *Cancer Nanotechnology*

**DOI:** 10.1186/s12645-017-0033-1

**Published:** 2017-11-10

**Authors:** Fred Currell, Steven Curley, Željka Krpetić, Mark Bellringer

**Affiliations:** 1 Queen’s University Belfast, Belfast, UK; 2 Baylor College of Medicine, Houston, USA; 3 University of Salford, Manchester, UK; 4 Mark Bellringer, Brighton, UK

Cancer is a group of diseases driven by inherently nanostructural problems (e.g. DNA issues). As such, there are obvious benefits to treatments employing nanoscale structures and processes. Additionally, as nanotechnologies are developing at a rapid rate, it is likely that many new themes will develop within the area in the next few years. With this in mind, *Cancer Nanotechnology* aims to provide a forum so that the most promising emerging themes should be pre-eminent in the minds of researchers working in one—or, indeed, both—of the disciplines of cancer research or nanotechnology.

*Cancer Nanotechnology* is published by SpringerOpen, a suite of fully open access journals that is part of the wider Springer Nature group. It enjoys a growing readership with an ever-increasing number of papers receiving double-digit citations.

As we near the end of another year in the life of *Cancer Nanotechnology*, it is worth reflecting upon the journal’s *raison d’être* and future direction.

The label is very much on the tin! *Cancer Nanotechnology* concerns itself with all that is new at the intersection of cancer research and nanotechnology. Relationships to cancer are deliberately interpreted broadly, encompassing: prevention, treatment, diagnosis, and related regulatory frameworks. Nanotechnology is taken to mean anything involving objects with characteristic length scales typically below 500 nm, but also includes the induction of nanoscale processes, for example by the injection of energy or various substances.

These definitions are intentionally wide, and the journal continues this approach: submissions accepted range from fundamental discovery and creation of basic techniques—including all phases of development—to the clinical application of nano-processes and technologies in relation to cancer treatment, diagnosis, and prevention.

One distinctive feature of the journal is the hosting of special thematic article collections; each collection serves to illustrate a research area’s present status and/or to highlight cutting-edge developments.

Such a collection acts as a vehicle which the research community can use to make important connections explicit and more widely disseminated. Furthermore, a collection comprising mainly review articles has the scope to show historical trend, whilst one focusing on original research can capture disruptive change. Of course, a collection can contain a mix of original research and reviews, offering the opportunity to trace both historical trend and innovative discontinuity. If there is a substantive development at the intersection of cancer and nanotechnology, then it needs a special thematic article collection—the journal’s editorial team welcomes your suggestions in this regard.

What is more, a key feature of collections is that they are ever evolving. As an online-only publication, *Cancer Nanotechnology* is not constrained by the rules of traditional special issues, the print analogy to our special collections. This allows the special collections to be dynamic—they can be updated as new, relevant, research is reported even several years after the first papers comprising the collection came together.

It is possible that any of the papers found in *Cancer Nanotechnology* could well have found an alternative venue for publication. But it would be much harder to find another specialist journal featuring the full diversity of this subject area. The intention is to create a unified forum for the full gamut of relevant research, thereby allowing developments and connections across the field to become apparent. We invite you to join us on our journey towards making this a reality.

